# Skin Disorders and Osteoporosis: Unraveling the Interplay Between Vitamin D, Microbiota, and Epigenetics Within the Skin–Bone Axis

**DOI:** 10.3390/ijms26010179

**Published:** 2024-12-28

**Authors:** Vincenzo Papa, Federica Li Pomi, Paola Lucia Minciullo, Francesco Borgia, Sebastiano Gangemi

**Affiliations:** 1 Department of Clinical and Experimental Medicine, School and Operative Unit of Allergy and Clinical Immunology, University of Messina, 98125 Messina, Italy; papavi.994@gmail.com (V.P.); sebastiano.gangemi@unime.it (S.G.); 2 Department of Precision Medicine in Medical, Surgical and Critical Care (Me.Pre.C.C.), University of Palermo, 90127 Palermo, Italy; federicalipomi@hotmail.it; 3 Department of Clinical and Experimental Medicine, Section of Dermatology, University of Messina, 98125 Messina, Italy; fborgia@unime.it

**Keywords:** skin-bone axis, osteoporosis, vitamin D, microbiota, microRNAs, miRNA, skin, psoriasis, atopic dermatitis, inflammatory skin diseases

## Abstract

Growing scientific evidence suggests a strong interconnection between inflammatory skin diseases and osteoporosis (OP), a systemic condition characterized by decreased bone density and structural fragility. These conditions seem to share common pathophysiological mechanisms, including immune dysregulation, chronic inflammation, and vitamin D deficiency, which play a crucial role in both skin and bone health. Additionally, the roles of gut microbiota (GM) and epigenetic regulation via microRNAs (miRNAs) emerge as key elements influencing the progression of both conditions. This review aims to examine the skin–bone axis, exploring how factors such as vitamin D, GM, and miRNAs interact in a subtle pathophysiological interplay driving skin inflammation and immune-metabolic bone alterations. Recent research suggests that combined therapeutic approaches—including vitamin D supplementation, targeted microbiota interventions, and miRNA-based therapies—could be promising strategies for managing comorbid inflammatory skin diseases and OP. This perspective highlights the need for multidisciplinary approaches in the clinical management of conditions related to the skin-bone axis.

## 1. Introduction

The interplay between cutaneous pathologies and systemic diseases is increasingly recognized, with the skin serving as a “signal” for internal conditions, including bone disorders. As the human body’s largest organ, the skin plays a pivotal role in endocrine regulation, immune response, and calcium-phosphorus metabolism, all of which intersect with bone health. Similarly, bone is not merely a structural framework but also a source of signaling molecules, such as osteocalcin and fibroblast growth factor 23, which influence skin physiology. Dysregulation of this interplay has been implicated in various pathological conditions, including osteoporosis and inflammatory skin disorders. The interconnected regulation systems of these tissues’ pathological changes support the concept of the “skin-bone axis,” suggesting that skin health may reflect bone health and vice versa [1].

Specifically, growing evidence suggests that patients affected by inflammatory cutaneous diseases, such as psoriasis and atopic dermatitis (AD), exhibit a heightened risk of suffering from bone comorbidities, including osteoporosis (OP), a systemic skeletal disorder characterized by reduced bone density and structural deterioration of bone tissue, which leads to increased fracture susceptibility [2]. The pathophysiological link between chronic skin inflammation and bone metabolism alteration appears to be multifactorial, involving immune dysregulation, systemic inflammation, and endocrine factors [1]. Immunologically, chronic skin conditions, such as psoriasis, are associated with the activation of T-helper (Th) 17 cells and the increased production of pro-inflammatory cytokines, such as interleukin (IL)-17, IL-1β, and tumor necrosis factor-α (TNF-α), which are known to promote osteoclastogenesis and enhance bone resorption. These cytokines increase the expression of the receptor activator of nuclear factor kappa-B ligand (RANKL), a key mediator in osteoclast differentiation, thereby contributing to the development of osteoporosis. Inflammatory factors also contribute to bone metabolism disruption by suppressing the Wnt/β-catenin signaling pathway, which is crucial for osteoblast differentiation and bone formation. Endocrine factors also play a significant role in this interplay [3,4,5]. Vitamin D, a key regulator of calcium and phosphorus homeostasis, which plays a pivotal role in bone health, also exerts immunomodulatory effects on the skin, thus influencing the severity and progression of inflammatory cutaneous conditions. Vitamin D deficiency is common among patients with cutaneous diseases and may contribute to the exacerbation of skin inflammation and concomitant OP onset [2]. Specifically, the role of vitamin D in modulating the immune response, particularly through its impact on T cells and cytokine production, suggests that it may serve as a critical mediator in the crosstalk between skin inflammation and bone resorption [2].

Another emerging factor in this complex interplay is the gut microbiome (GM), which is increasingly recognized as a central player in skin and bone health. Dysbiosis, namely the imbalance in the GM, has been implicated in the pathogenesis of cutaneous diseases, influencing immune responses and systemic inflammation. Moreover, alterations in GM composition have been linked to bone homeostasis alterations through the dysregulation of nutrient absorption, including vitamin D and calcium, and the modulation of osteoclast activity. For example, *Faecalibacterium prausnitzii* is typically diminished in both inflammatory skin diseases and osteoporosis, with reduction being associated with increased inflammation and disrupted immune regulation, thus highlighting its protective role in systemic health. This bidirectional communication between the gut, skin, and bone underscores the role of the microbiota in understanding the intricate crosstalk of these seemingly so-different diseases [6,7].

Finally, microRNAs (miRNAs), small non-coding RNAs that play an epigenetic role through the regulation of gene expression, are emerging as key regulators in both inflammatory cutaneous diseases and OP. MiRNAs seem to modulate inflammatory pathways, immune cell differentiation, and bone remodeling processes, suggesting a potential role in linking chronic skin inflammation with bone degradation. Specifically, miR-21 has been associated with the hyperproliferation of keratinocytes in psoriasis and the regulation of bone remodeling, influencing osteoclast activity, while miR-31 is involved in cutaneous keratinocyte differentiation while modulating osteoblast differentiation and influencing bone formation. Alterations in miRNA expression profiles in inflammatory cutaneous disorders and OP patients point to their possible role as disease biomarkers and potential therapeutic targets [8].

This narrative review aims to explore the intricate interplay among vitamin D, GM, and miRNAs in chronic inflammatory/autoimmune skin diseases and OP. In the context of an emerging bidirectional communication within the skin–bone axis, our analysis paves the way for future research aimed at a more detailed characterization of overlapping cytomolecular pathways, which are essential for elucidating novel potential combinatory therapeutic approaches within a renewed multidisciplinary setting.

## 2. Materials and Methods

We conducted a comprehensive search in the PubMed electronic database, applying no temporal or study type restrictions but maintaining a mechanistic focus on relevant English-language studies published within the last decade. As keywords, the three core elements of our narrative review—“vitamin D,” “microbiota,” and “miRNA”—were individually matched with “osteoporosis” and the various inflammatory/autoimmune dermopathies discussed.

## 3. Discussion

### 3.1. Chronic Inflammatory and Autoimmune Skin Diseases

#### 3.1.1. Role of Vitamin D

Vitamin D is a pro-hormone that plays a key role in immune response regulation, with both immunomodulatory and anti-inflammatory effects. The primary sources of vitamin D are exogenous dietary intake and endogenous production in the skin through sunlight exposure. Ergocalciferol (vitamin D₂) is derived from the ultraviolet (UV) irradiation of ergosterol, while epidermal 7-dehydrocholesteyrol, or provitamin D₃, absorbs UV rays and converts into previtamin D₃. This conversion is essential for producing the biologically active form, 1,25-dihydroxyvitamin D (1,25(OH)2D), namely calcitriol. Previtamin D₃ is subsequently converted into vitamin D, which undergoes hydroxylation reactions in the liver and kidneys [9]. Its activity is mediated through the vitamin D receptor (VDR), which is expressed on various immune cells, such as T cells, dendritic cells, and keratinocytes. Upon activation, vitamin D binds to the VDR and modulates the transcription of genes involved in immune function, cell proliferation, and skin barrier integrity. Several researchers have found associations between low vitamin D levels and multiple autoimmune and inflammatory diseases, including psoriasis, AD, and vitiligo. Specifically, in psoriasis, a correlation between low serum levels of 25(OH)D and disease severity has been highlighted. Vitamin D, in its active form calcitriol, binding to VDR expressed in keratinocytes and immune cells, regulates gene expression and modulates inflammatory pathways. This interaction inhibits keratinocyte hyperproliferation, promotes proper differentiation, and restores skin cell turnover. Additionally, vitamin D suppresses Th1- and Th17-mediated immune responses by reducing pro-inflammatory cytokines, including IL-17, IL-22, and interferon-γ (IFN-γ), while enhancing regulatory T cell activity to restore immune tolerance. It also decreases the production of inflammatory mediators like TNF-α and IL-23, while promoting anti-inflammatory cytokines such as IL-10. Furthermore, vitamin D contributes to skin barrier integrity by upregulating proteins like filaggrin and involucrin, addressing a common deficit in psoriasis [10,11]. A systematic review and meta-analysis confirmed this inverse relationship, showing that psoriatic patients had significantly lower serum 25(OH)D compared to healthy controls [10]. However, other findings from population-based studies challenge this relationship by showing no significant deficiency, underlining the impact of variability in diet, race, and UV exposure on 25(OH)D levels [12,13]. Moving from this evidence, clinical trials have studied the efficacy of vitamin D supplementation in reducing psoriasis severity. Finamor et al. reported a significant reduction in Psoriasis Area Severity Index (PASI) scores following six months of high-dose vitamin D3 treatment [14]. Furthermore, topical vitamin D analogs, such as calcipotriol and tacalcitol, have demonstrated excellent efficacy, especially when combined with corticosteroids or phototherapy, having now become the first-line treatment for mild to moderate psoriasis [15]. Mechanistically, vitamin D inhibits Th17-driven inflammation and suppresses pro-inflammatory cytokines like IL-12/23 and TNF-α, which are overexpressed in psoriasis, thus improving skin barrier function by regulating proteins such as psoriasin and late cornified envelope proteins [16].

Moving to AD, the relationship between vitamin D deficiency and AD pathogenesis is more complex, with inconsistent findings across studies. Nevertheless, most of the existing research suggests a link between vitamin D and both the risk and severity of atopic dermatitis [17]. In a mouse model of AD induced by ovalbumin, vitamin D significantly improved skin condition, reduced IgE and IL-5 levels, increased IL-4 and IL-13 levels, lowered filaggrin expression, and decreased epidermal thickness. Histological analysis further confirmed that vitamin D effectively alleviates inflammation and improves the pathological state of the skin [18]. Epidemiological research shows higher prevalence rates of AD in regions with limited sun exposure, suggesting a potential role for vitamin D deficiency in disease onset [19]. In parallel with what has been observed in psoriasis, some clinical studies have highlighted that patients with low serum 25(OH)D levels experience more severe AD symptoms, with higher SCORing Atopic Dermatitis (SCORAD) scores reflecting disease severity [20].

Confirming this evidence, vitamin D supplementation has shown potential benefits in AD by modulating immune responses, restoring the Th1/Th2 cytokine balances, improving skin barrier function, and reducing the pro-inflammatory IL-4, IL-6, and IFN-γ [21,22]. In addition to vitamin D oral supplementation, phototherapy, through exposure to UV rays, performs a dual function: it increases 25(OH)D serum levels while creating immunomodulatory effects, reducing both adaptive and innate immune responses. This immune modulation decreases pro-inflammatory cytokine production while boosting IL-10 levels, which plays an immunosuppressive role. Additionally, UV exposure strengthens the skin’s structural integrity and function by thickening the stratum corneum, which enhances barrier protection and helps reduce eczema flare-ups. Finally, vitamin D also increases antimicrobial peptides (AMPs) such as LL-37, which reduces *Staphylococcus (S.) aureus* colonization and prevents complications like eczema herpeticum [21]. 

Vitamin D also plays a key role in vitiligo by regulating immune responses, supporting melanocyte function, and reducing oxidative stress (OS). In vitiligo, melanocytes are targeted and destroyed by the cluster of differentiation (CD)8+ T cells, driven by inflammatory cytokines like IL-2, IFN-γ, and TNF-α. Calcitriol, the active form of vitamin D, binds to VDR on immune cells, reducing inflammation by downregulating these cytokines and enhancing regulatory T cell (Treg) function, thus protecting melanocytes from autoimmune destruction. Additionally, vitamin D promotes melanin production by upregulating microphthalmia-associated transcription factor (MITF), which increases tyrosinase activity—essential for melanin synthesis—thereby encouraging repigmentation. Moreover, vitamin D reduces reactive oxygen species (ROS) levels by activating antioxidant pathways, thereby protecting melanocytes from OS. Vitamin D further supports melanocyte survival through the release of growth factors such as stem cell factor (SCF), which binds to the c-KIT receptor to promote melanocyte growth. These combined effects of vitamin D suggest its potential as a therapeutic agent in vitiligo, aiding both repigmentation and stabilization of the autoimmune and oxidative conditions that contribute to melanocyte loss [23]. Moreover, evidence suggests that vitamin D supplementation could help restore immune balance by downregulating pro-inflammatory cytokines, which may slow the progression of vitiligo-related depigmentation [24].

Similarly, hypovitaminosis D is associated with alopecia areata (AA), a chronic T-cell-mediated autoimmune disorder whose pathogenesis involves the loss of immune privilege in hair follicles, driven by autoreactive T cells, mast cells (MCs), CD8+ NKG2D+ cytotoxic T cells, and the Janus kinase/signal transducer and activator of transcription (JAK/STAT) pathway [25]. Specifically, hair follicles are normally considered immune-privileged sites, primarily due to the downregulation of major histocompatibility complex (MHC) class I and class II molecules, limited expression of costimulatory molecules, and active secretion of immunosuppressive cytokines such as transforming growth factor-β (TGF-β) and IL-10. This immune privilege protects hair follicles from immune-mediated damage, particularly during the anagen phase. In genetically predisposed individuals, autoreactive CD8+ T cells play a pivotal role in the loss of immune privilege. Cytokines, particularly IFN-γ, secreted by activated CD4+ helper T cells and other immune cells, induce the upregulation of MHC class I and II molecules on follicular keratinocytes and melanocytes. This upregulation exposes hair follicle antigens to the immune system, marking them as targets for autoreactive T cells. The recruitment of CD8+ NKG2D+ cytotoxic T cells is especially significant. These cells recognize stress-induced ligands, such as MICA and MICB, which are overexpressed on hair follicle cells under inflammatory conditions, further driving the cytotoxic response. The breakdown of immune privilege is perpetuated by the release of pro-inflammatory cytokines, including IL-15, IL-2, and IFN-γ, which amplify T-cell activation and effector functions. MCs and other innate immune cells contribute to this process by releasing histamine and cytokines that facilitate the recruitment and activation of T cells in the follicular microenvironment [26,27]. Vitamin D appears to counter several pathogenic mechanisms in AA. It reduces IFN-γ production and CD8+/CD4+ T-cell cytokine activity, helping maintain immune privilege in hair follicles. Additionally, vitamin D stabilizes MCs, which otherwise promote autoantigen presentation to CD8+ T cells and increase proinflammatory signaling. Moreover, it downregulates key activating ligands, including C-X-C motif chemokine ligand 10 (CXCL-10), for CD8+ NKG2D+ T cells, reducing their activation and trafficking, and inhibits the JAK/STAT pathway, attenuating the action of major AA-related cytokines such as IL-2, IL-7, IL-15, IL-21, and INF-γ. Confirming the crucial role of vitamin D, studies have reported significant improvement with calcipotriol applications through reduction of epidermal cell proliferation, promotion of keratinocyte differentiation, and modulation of cytokine production [28]. Systematic reviews have highlighted an inverse relationship between serum vitamin D levels and AA severity, thus suggesting that screening for vitamin D deficiency and vitamin D supplementation may be beneficial in the treatment of patients with AA [29,30]. However, further research is required to confirm these findings [31].

Concerning autoimmune blistering skin disorders, the significantly low serum levels of 25(OH)D in patients with pemphigus vulgaris compared to healthy controls, as well as the negative correlation between vitamin D levels and disease severity, would suggest a presumed pathogenic role of hypovitaminosis D in the disease. Mechanistically, vitamin D acts as a critical immunomodulator in the skin. Under physiological conditions, it enhances innate immune responses against infections and supports tissue repair and wound healing by activating toll-like receptor 2 (TLR2). Additionally, vitamin D promotes the expansion of Tregs and Th2 cytokines while downregulating Th1 and Th17 cytokines. In pemphigus vulgaris, Tregs play a pivotal role in controlling the activity of desmoglein-3-reactive lymphocytes, thereby preventing disease onset. Consequently, vitamin D deficiency and the associated impairment of Treg activity represent a significant etiopathogenic link in this dermopathy [32]. More recent experimental evidence in a mouse model of epidermolysis bullosa acquisita would confirm the immunomodulatory role of vitamin D via increase of CD4+ forkhead box P3 (FoxP3)+ Tregs and B cells (CD19 + IL10+), concomitant with the reduction of proinflammatory Th17 cells [33].

#### 3.1.2. Role of Gut Microbiota

The critical role of GM in modulating immune responses and influencing several cutaneous disorders through the so-called “gut–skin axis”, namely the bidirectional interplay between the gastrointestinal tract and the skin, has become a focus of research, especially in understanding inflammatory skin diseases [34,35]. This relationship is mediated by an impaired intestinal barrier, heightened inflammatory mediators, and microbial metabolites that impact skin health. When the GM is disturbed, or its interface with the host is altered, it can trigger immune responses that in turn increase susceptibility to pathogens. Such imbalances in the microbiome, namely dysbiosis, can lead to both local and systemic inflammation, ultimately raising the risk of developing systemic diseases that may have skin-related manifestations [36]. In psoriasis, gut dysbiosis has been linked to chronic systemic inflammation driven by Th17 cells, which promote keratinocyte hyperproliferation [36]. Specifically, gut dysbiosis leads to a weakened intestinal barrier, often referred to as a “leaky gut,” which allows the translocation of microbial antigens, metabolites, and lipopolysaccharides (LPS) into the systemic circulation. These microbial products act as potent stimulators of the immune system, activating antigen-presenting cells (APCs) such as dendritic cells in the gut-associated lymphoid tissue (GALT). APCs, in response to microbial components, secrete cytokines like IL-6, IL-1β, and TGF-β, which are critical for the differentiation of naïve CD4+ T cells into Th17 cells. Th17 cells are central players in the psoriatic inflammatory cascade, producing pro-inflammatory cytokines such as IL-17A, IL-22, and IL-23. These cytokines not only sustain the activation of Th17 cells but also promote keratinocyte proliferation and the recruitment of neutrophils and other immune cells to the skin [37,38]. Moreover, a reduction in beneficial bacteria such as *Faecalibacterium* and *Akkermansia muciniphila* can impair the production of short-chain fatty acids (SCFAs) like butyrate, which regulates immune function by inhibiting the activation of pro-inflammatory pathways, including those involving Th17 cells [39,40]. Disruptions in microbial balance also impair the gut barrier, promoting systemic inflammation and allowing microbial products like LPS to enter circulation, triggering the release of inflammatory cytokines, such as TNF-α, IL-17, and IL-23, that exacerbate psoriatic plaques [34,35,36,41]. Furthermore, studies have highlighted that psoriasis severity correlates with decreased bacterial diversity and concomitant increased levels of pro-inflammatory bacteria such as *Escherichia (E.) coli* and *Ruminococcus gnavus* [42]. However, probiotics and dietary interventions targeting gut health are showing potential in alleviating psoriasis symptoms, modulating gut microbiota, reducing systemic inflammation, and improving skin barrier function. For instance, supplementation with *Bifidobacterium* and *Lactobacillus* species has been reported to reduce PASI scores by modulating systemic immune responses. A randomized controlled clinical trial investigated the efficacy and safety of oral administration of a mixture of probiotic strains, including *Bifidobacterium* and *Lactobacillus*, in patients with moderate to severe psoriasis, thus finding a significant reduction in PASI scores up to 75% in the probiotic group compared to the placebo group (66.7% vs. 41.9%; *p* < 0.05) [43]. *Lactobacillus paracasei*, for example, has been found to reduce transepidermal water loss (TEWL) and enhance barrier integrity, while *Bifidobacterium infantis* showed reductions in pro-inflammatory markers, including TNF-α, highlighting their potential in managing psoriasis severity and patients’ quality of life. However, the exact mechanisms linking specific gut bacteria to psoriasis progression remain under investigation.

Similarly, in AD, gut dysbiosis has been linked to impaired immune balance, particularly through disruptions in Th1/Th2 immune responses. Recent investigations have highlighted that AD patients often exhibit reduced levels of beneficial gut bacteria, including *Lactobacillus* and *Bifidobacterium*, along with decreased microbial diversity [44].

This microbial imbalance can lead to compromised gut barrier function, promoting systemic immune activation and increasing susceptibility to allergens. Furthermore, altered microbial metabolites, including SCFAs, seem to influence the expression of pro-inflammatory cytokines like IL-4, IL-5, and IL-13, which are hallmarks of AD [45]. Probiotics and prebiotics have been explored as therapeutic interventions for AD. Studies suggest that supplementation with *Lactobacillus rhamnosus* can alleviate AD symptoms by modulating immune responses and restoring microbial balance [46]. Additionally, symbiotic interventions combining probiotics and prebiotics have been shown to reduce the severity of eczema and lower SCORAD scores in children [47]. However, results remain inconsistent, with some studies reporting limited or no benefit, highlighting the need for further research on optimal probiotic strains and doses.

In vitiligo, gut dysbiosis, characterized by reduced *Bacteroidetes* and *Lachnospiraceae*, seems to be able to increase OS and ROS production, thus promoting immune dysfunction and melanocyte destruction. A recent study emphasizes that patients with vitiligo exhibit altered gut microbial profiles, which may impair immune tolerance and melanocyte survival, further driving disease progression [24]. From this premise, it emerges that targeting GM could be a novel therapeutic strategy for vitiligo: reestablishing microbial diversity might mitigate autoimmunity and promote repigmentation.

Research suggests a link between gut dysbiosis and alopecia areata (AA), with parallels seen in gut microbiome-related conditions like inflammatory bowel disease (IBD). IBD dysbiosis, marked by reduced *Bifidobacteria* and elevated *Bacteroides*, *Enterobacteriaceae*, *Erysipelotrichaceae* and *Clostridiales*, is associated with an increased prevalence of AA, particularly in ulcerative colitis and Crohn’s disease, suggesting shared inflammatory pathways [48,49]. In both AA and IBD, elevated Th1 cytokines like TNF-α may play a role, though it remains unclear if common microbial changes are involved. A study on alopecia universalis found no major differences in microbiome diversity compared to healthy controls but identified increased levels of specific bacteria (e.g., *Holdemania filiformis*, *Erysipelotrichaceae*), some of which are linked to inflammatory responses, while another study identified higher levels of *Bacilli* and *Lactobacillales* in AA patients, pointing to possible immune-modulatory effects from GM in AA. Therapies targeting the gut–skin axis are showing promise, though results vary [50]. For instance, SCFA propionate treatment in mice yielded inconsistent hair regrowth, while fecal microbiota transplants have led to hair growth improvement in patients with AA alongside treating gut infections like *Clostridium difficile* [51]. Additionally, vitamin D deficiency, often linked to AA, may impair gut microbiota regulation and reduce beneficial SCFAs, potentially impacting hair health [52]. Emerging treatments include a platelet-rich plasma-like gel containing postbiotics, which showed efficacy in improving AA severity, and probiotics, which increased the Treg/CD4+ ratio in lymph nodes [53]. These findings underscore the GM potential role in AA and warrant further exploration into microbial therapies for AA.

Recent in-depth studies on autoimmune blistering diseases have highlighted significant GM alterations in patients with bullous pemphigoid. These patients exhibit reduced gut microbial diversity (alpha diversity) and marked dysbiosis, characterized by an increase in *Bacteroidaceae*, *Ruminococcaceae*, and *Enterobacteriaceae*, along with a decrease in *Lachnospiraceae*, *Prevotellaceae*, and *Veillonellaceae*. Notably, a reduction in *Faecalibacterium prausnitzii*—a trait also observed in the gut microbiomes of AD and psoriasis patients—has been identified as a distinguishing feature. Additionally, an overexpression of gamma-aminobutyric acid (GABA)-mediated pathways was found, underscoring unique pathophysiologic mechanisms in these patients. Beyond its recognized antioxidant and anti-inflammatory roles, GABA contributes to skin health through various mechanisms. As a neurotransmitter, it inhibits itch signaling; as an immunomodulator, it helps maintain the Th1/Th2 balance; and structurally, it preserves skin elasticity by promoting increased type 1 collagen expression. In patients with bullous pemphigoid, an enrichment of the GM-derived GABA shunt has been observed. This pathway involves the conversion of L-glutamate into GABA, followed by the conversion of GABA into succinate. Specifically, increased biosynthesis of pyridoxal 5′-phosphate—a key cofactor in the GABA shunt responsible for converting GABA into succinate—has been reported, alongside elevated biosynthesis of putrescine, a precursor of GABA. Interestingly, putrescine has already been positively associated with disease severity in AD patients [54,55].

In conclusion, GM seems to influence the pathogenesis of inflammatory skin diseases by modulating immune and inflammatory pathways. These findings support the development of gut-targeted interventions, such as dietary modifications and probiotics administration, to restore microbial homeostasis and reduce disease severity.

#### 3.1.3. Role of miRNAs

The increasing understanding of the molecular regulatory network involving miRNAs in inflammation-associated skin diseases is paving the way for new diagnostic and therapeutic strategies. These strategies utilize miRNAs as disease biomarkers and employ miRNA inhibitors/antagomirs and miRNA mimics/agomirs. To date, in the context of chronic dermatoses, most available evidence remains preliminary, encompassing both in vitro and in vivo studies, inspired by some clinical trials primarily conducted on patients with psoriasis and AD. In psoriasis, the delivery of miR-99a mimics to keratinocytes (HaCaT) resulted in the suppression of keratinocyte proliferation via miR-99a overexpression, as this microRNA is downregulated in psoriatic lesions. Similarly, transfection with miR-125a mimics into HaCaT cells achieved the same outcome. Furthermore, intradermal injection of synthetic miR-146a into wild-type mice with psoriasiform dermatitis led to miR-146a overexpression (which is downregulated in psoriasis), resulting in a reduction in erythema, epidermal thickness, scaling, and neutrophil infiltration. In AD, transfection of miR-124 mimics into keratinocytes increased miR-124 expression (downregulated in lesional skin of AD patients), thereby modulating NF-κB-associated inflammatory pathways in activated keratinocytes. Similarly, transfection of miR-10a-5p (upregulated in AD) mimics into IL-1β-stimulated keratinocytes inhibited keratinocyte proliferation. These promising strategies are not without practical challenges that remain to be addressed, including the identification of an appropriate administration route and an efficient delivery system, targeting specific cells, ensuring intracorporeal stability, and achieving the desired intracellular effects [56]. The dysregulation of miRNAs plays a crucial role in the pathogenesis of psoriasis, as they are involved in the regulation of keratinocyte hyperproliferation and abnormal differentiation, as well as in dysfunctional immune responses and epidermal inflammation. Among the miRNAs implicated in the regulation of inflammation and keratinocyte proliferation are nuclear factor kappa-light-chain-enhancer of activated B cells (NF-κB) activation-related miR-31 and the IL-17A and NF-κB dual-regulated miR-146a. Specifically, miR-146a is upregulated by NF-κB during the late stages of inflammation, and as part of a negative feedback mechanism, it limits NF-κB signaling [55]. Similarly, tissue levels of miR-9 are significantly reduced in psoriasis patients, suggesting a potential anti-inflammatory role for miR-9 through a feedback mechanism that downregulates NFKB1. Indeed, miR-9 is upregulated by NFKB itself via LPS and myeloid differentiation primary response 88 (MyD88) interactions. Interestingly, pro-inflammatory cytokines that are upregulated in psoriasis (such as TNF-α, Toll-IL-1R (TIR), and IL-1β) also upregulate miR-9 [57].

Regarding miR-99a, which is downregulated in psoriasis, a functional role is emphasized in the downregulation of insulin-like growth factor 1 receptor (IGF1R) signaling, which is upregulated in psoriasis and is responsible for hyperkeratosis and epidermal hyperplasia. Conversely, in the modulation of psoriatic immune dysfunction, miR-138 (downregulated in psoriasis) plays a role in regulating the Th1/Th2 balance, while Th17 cell differentiation-related NF-κB-dependent miR-155 and miR-210 are responsible for both the loss of immunosuppressive function in Tregs by targeting FOXP3 and the induction of Th17 and Th1 cell differentiation while simultaneously inhibiting Th2 cell differentiation [55]. A significant pathogenic role is also attributed to the communication between keratinocytes and serum extracellular vesicles, which contributes to epidermal hyperplasia and skin inflammation in psoriasis through loaded miRNAs such as miR-1305 and miR-6785-5p [56,58]. Additionally, other miRNAs (such as miR-223), found to be upregulated in psoriasis, positively correlate with disease progression and activity [59]. Recently, Solvin et al. provided a comprehensive overview of the miRNA profile in psoriatic skin, identifying a miRNA signature of 11 miRNAs associated with disease severity. Furthermore, the differential expression of 20 miRNAs between control skin and non-lesional psoriatic skin suggests systemic immune dysregulation, indicating systemic inflammation based on shared molecular mechanisms between psoriasis and its related comorbidities [60].

An even more intriguing aspect is the recently detected high similarity between the miRNA expression profiles of perilesional skin in adult patients with AD and psoriasis. Specifically, miR-28-5p, miR-31-5p, miR-378a-3p, and miR-203a were validated as upregulated and likely involved in regulating immune responses in both skin conditions [61]. Abdallah et al. recently evaluated the expression profile of 11 circulating immune-related miRNAs (miRNA-7, miRNA-9, miRNA-23b, miRNA-124, miRNA-145, miR148a, miRNA-148b, miRNA-155, miRNA-181a, miRNA-203a, and miRNA-320a) in psoriasis and vitiligo, finding them to be upregulated in both skin disorders (more so in psoriasis), suggesting the existence of shared target genes and pathways [62]. Interestingly, specific miRNA polymorphisms in vitiligo may act as both protective factors (as observed for miR-211 rs8039189) or risk factors (as observed for miR-202 rs12355840) for disease susceptibility [63].

Recent literature reviews have extensively highlighted the functional dichotomy of the most commonly aberrant miRNAs expressed in chronic autoimmune and inflammatory skin disorders. For example, miR-21 is upregulated in psoriasis (involved in T-cell activation, inflammation, stimulation of keratinocyte proliferation, and inhibition of apoptosis) and downregulated in vitiligo (with a melanoprotective role via modulation of Treg/Teff balance). MiR-211, downregulated in vitiligo, also has a protective role, as it promotes pigmentation in melanocytes and melanoblasts. Conversely, miR-25, miR-9, miR-377, and miR-493-3p (upregulated in vitiligo) have a presumed pathogenic role, as they promote OS mechanisms in melanocytes, keratinocytes, and other cell types. Additionally, miR-155, similar to its role in psoriasis, is pro-inflammatory in vitiligo [64,65].

In AD, a recent systematic review has underscored the crucial pathogenic role of miRNA dysregulation involved in the regulation of keratinocyte apoptosis/proliferation, NF-κB-dependent inflammation, and Th17 and Treg immune activity. Notably, aberrant upregulation of miR-10a-5p and miR-29b contributes to AD-associated epithelial barrier dysfunction by worsening the proliferation or inducing the apoptosis of keratinocytes. A crucial pathogenic role in AD is ascribed to miR-155 (one of the most upregulated miRNAs in AD patients) through its action promoting Th17 differentiation. In contrast, miR-124 (downregulated in AD) has a recognized anti-inflammatory role by inhibiting p65, a subunit of NF-κB, as evidenced by the strong inhibitory effect that TNF-α exerts on it. Similarly, miR-146a (upregulated in AD) functions as an anti-inflammatory miRNA by targeting upstream mediators of NF-κB signaling—IL-1 receptor-associated kinase 1 (IRAK1) and Caspase Recruitment Domain Family Member 10 (CARD10), in addition to limiting type-2-cell-mediated immune responses. Another crucial anti-inflammatory role in AD is recognized for miRNA-143, which targets interleukin 13 receptor alpha 1 (IL-13Ra1) in epidermal keratinocytes [66].

A recently investigated etiopathogenic link between epigenetics and the skin microbiome in AD involves miR-939, suggesting a crucial regulatory role through increased expression of matrix metalloproteinase (MMP)1, MMP3, MMP9, and Intercellular Adhesion Molecule 1 (ICAM1) in human keratinocytes, promoting *S. aureus* colonization and the subsequent exacerbation of *S. aureus*-induced AD-like skin inflammation [67]. Alongside the identification of specific miRNAs (such as miR-101) as potentially valuable diagnostic biomarkers, the expanding understanding of the regulated molecular pathways, as well as the manipulation of dysregulated miRNAs (especially in Tregs), is opening new therapeutic avenues even in AA [68,69,70,71]. Mustafa et al. recently found significant upregulation of miRNAs-203, 146a, and 155 in the lesional tissue of AA patients compared to controls, with significantly higher expression levels in active disease compared to the inactive form, suggesting their crucial pathogenic involvement as well as their being potential indicators of disease activity [72]. Moreover, in severe active AA, a potential functional pathogenic synergy is observed among the dysregulated circulating miRNAs miR-185-5p, miR-125b-5p, and miR-186-5p [73].

**Figure 1:**
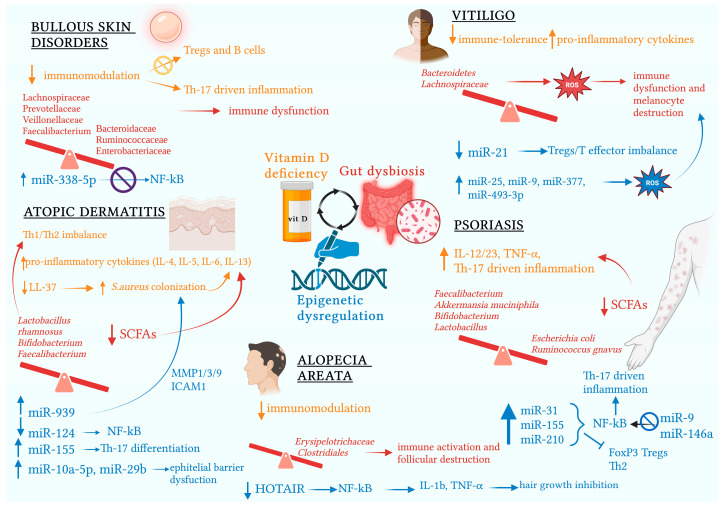
Representation of the key etiopathogenic role played by the interplay between vitamin D deficiency, gut dysbiosis (resulting in reduced SCFAs production), and miRNA dysregulation in the onset of major chronic inflammatory and/or autoimmune skin disorders. To optimize visualization for each condition, pathways related to key pathophysiological mechanisms are highlighted in three distinct colors: yellow for vitamin D deficiency, red for gut dysbiosis, and blue for epigenetic dysregulation. Down arrows indicate a reduction, while up arrows indicate an increase. Additionally, for the various conditions depicted, within partially overlapping dysbiotic profiles and distinct epigenetic dysregulation patterns, the key pathophysiological events are largely attributable to impaired immune regulation and consequent chronic inflammation. Abbreviations: forkhead box P3 regulatory T cells (FoxP3 Tregs), SCFAs (short chain fatty acids), reactive oxygen species (ROS), interleukin (IL), tumor necrosis factor α (TNFα), nuclear factor kappa-light-chain-enhancer of activated B cells (NF-κB), matrix metalloproteinases (MMPs), Intercellular Adhesion Molecule 1 (ICAM1). Created with Biorender.com.

MiR-205 is also crucial for pathogenesis, with significantly elevated serum levels found in AA patients, playing an important role in modulating immune responses and promoting the transition of hair follicles from growth to regression and resting phases. Conversely, serum levels of the long non-coding (lnc) RNA HOTAIR were found to be significantly downregulated in AA patients. This could explain the crucial pathogenic involvement of HOTAIR deficiency in AA, given its substantial role in suppressing NF-κB-related inflammation by downregulating IL-1β and TNF-α, potent cytokines inhibiting hair growth [74]. In the context of autoimmune blistering diseases, miR-338-3p is one of the most overexpressed miRNAs in pemphigus, with a downregulatory role in the NF-κB-related pathway. Other miRNAs overexpressed in pemphigus that are potentially pathogenic through their regulatory actions on various signaling pathways include miR-424-5p, miR-584-5p, and miR-326. Similarly, in bullous pemphigoid, the overexpression of miR-1291, miR-27a-5p, and miR-423-5p suggests a significant pathogenic, diagnostic, and prognostic role [75]. Overall, these findings underline the potential of utilizing miRNAs as biomarkers and therapeutic targets, emphasizing the need for further exploration of the miRNA molecular network in chronic inflammatory skin diseases [64] (Figure 1).

### 3.2. Osteoporosis

#### 3.2.1. Role of Vitamin D

In its classic definition, OP is a “skeletal disorder characterized by compromised bone strength, predisposing a person to an increased risk of fracture”. This definition encapsulates the two hallmark clinical features of the disease: (1) a pathological decrease in bone mineral density (BMD); (2) increased bone fragility as a direct consequence of qualitative and quantitative organ alterations.

The current World Health Organization (WHO) Expert Committee classification distinguishes between OP, defined by a BMD < −2.5 standard deviation (SD) T-score, and established OP, characterized by a BMD < −2.5 SD T-score + fragility fractures. This increased incidence of fractures is closely linked to the process of skeletal aging [76,77]. As an age-related phenomenon, it is responsible for the imbalance between osteogenesis and osteoclastogenesis, resulting in increased bone loss. The underlying pathophysiological mechanism is “inflammaging”. This refers to a persistent, low-grade, and gradually increasing systemic pro-inflammatory response occurring during the aging process as a natural consequence of extensive immune system remodeling throughout the lifespan. Specifically, the cyto-molecular basis of inflammaging is characterized by a qualitative and quantitative age-related decline in immune cells and the associated cytokine networks. This process is also reflected in the significant differences in the expression of immunological biomarkers observed between young and elderly individuals [78,79].

In its classical etiopathogenetic interpretation referring unilaterally to the endocrine model, OP recognizes as primary causal factors certain hormonal phenomena, such as estrogen deficiency, secondary hyperparathyroidism, hyperthyroidism, hyperprolactinemia, hypercortisolism, inadequate calcium intake, and vitamin D deficiency [76,77]. Vitamin D is essential for stimulating intestinal calcium and phosphorus absorption, and it plays a key hormonal role in controlling the bone mineralization process, which consists of restoring the balance between bone resorption and formation, thereby ensuring the maintenance of optimal bone microarchitecture [80,81,82].

Beyond its well-known hormonal function, vitamin D has an immunoregulatory role, and when deficient, it is equally crucial to the disease’s pathogenesis.

The 20-year-old concept of “osteoimmunology” has revolutionized the understanding of the pathophysiological mechanisms governing OP. Bone homeostasis is regulated by a well-defined pool of cytokines and immune cells. Both innate and adaptive immunity are involved in this metabolic regulation. Specifically, macrophages, B cells, natural killer (NK) cells, innate lymphoid cells (ILC)1, ILC3, Th-1, and Th-17 cells are actively involved in inducing osteoclastogenesis through the release of various pro-inflammatory cytokines such as RANKL, IL-1β, IL-8, IL-15, IL-17, and TNF-α. On the other hand, macrophages (thus having a dual functional role), Tregs, Th2 cells, and ILC-2 are essentially involved in suppressing osteoclastogenesis through the release of anti-inflammatory cytokines such as IL-10, IL-4, IL-5, and IL-13 [83].

Both due to the phenomenon of inflammaging and the potential inflammatory contribution from the coexistence of other chronic inflammatory conditions, OP should be considered an inflammatory pathology. In this condition, the dysregulation of bone immune homeostasis is characterized by an imbalance towards pro-inflammatory osteoclastogenic cytokines, which, when upregulated, contribute to bone resorption [83,84,85,86,87].

At the molecular level, immune-mediated bone loss would recognize the P2X7 receptor signaling as a key mediator, positioning this receptor as a critical interface between inflammatory response and bone turnover regulation. P2X7 is a component of the multiprotein complex known as the inflammasome and is highly expressed on the surface of various immune cells, especially macrophages. Its activation depends on high extracellular concentrations of adenosine triphosphate (ATP), such as those released in inflammatory settings. Once activated, the NLR family containing 3 (NLRP3) inflammasome, together with caspase-1 and caspase-3, stimulates the release of pro-inflammatory cytokines that contribute to the bone loss process. Furthermore, the expression of this receptor on osteoclasts has been extensively investigated, with the highest expression found in mature osteoclasts. In these cells, among the many intracellular pathways influenced, P2 × 7 leads to the activation of NFκB, which is a transcription factor essential for osteoclast development, along with the activation of Ca2+/calcineurin/nuclear factor of activated T cells c1 (NFATc1) signaling and increased expression of autophagy-related proteins during osteoclast differentiation [85,88,89]. Far beyond the mechanistic effects associated with P2 × 7 and NLRP3 inflammasome activation, immune-mediated bone loss is predominantly driven by the downstream actions of various pro-inflammatory cytokines mentioned above. Among these, RANKL is the primary molecular driver of osteoclastogenesis, functionally supported by other pro-inflammatory cytokines that promote its expression, including IL-1β, IL-7, IL-8, IL-15, IL-17, and TNF-α. RANKL, either secreted or expressed by both immune and skeletal cells, binds to its receptor RANK on the osteoclast surface. Upon formation of the RANK–RANKL complex, TNF receptor-associated factor 6 (TRAF6) is recruited on the cytoplasmic side, triggering downstream activation of multiple signaling pathways, such as inhibitory kappa B kinase (IKK) complexes, mitogen-activated protein kinases (MAPKs), and Ca2+/calmodulin-dependent protein kinase IV (Ca2+/CAMKIV)-cyclic adenosine monophosphate response element-binding protein (CREB). Collectively, these signaling cascades drive the translocation and activation of NFATc1, a key transcription factor required for the initiation of osteoclastogenic gene expression. Simultaneously, the activation of other critical signaling pathways, such as JAK/STAT, phosphatidylinositol 3-kinase (PI3K), and NF-κB—mediated by IL-6, macrophage colony-stimulating factor (M-CSF), and TNF-α, respectively—is equally essential in promoting osteoclast maturation, differentiation, and survival [83].

Vitamin D plays a key role in controlling systemic inflammation and OS, two major biological processes related to aging in humans. The anti-inflammatory role of 1,25(OH)2D (calcitriol) is primarily attributed to its inhibitory effect on the transcription factor NF-κB, which is deeply involved in promoting OS and chronic diffuse somatic inflammation. Consequently, vitamin D deficiency is directly implicated in the development and severity of various age-related metabolic disorders associated with OS, including OP [90].

Previous in vivo and in vitro studies have suggested the key role of vitamin D in its active form, 1,25-dihydroxyvitamin D (1,25[OH]2D3), in downregulating pro-inflammatory osteoclastogenic cytokines., Through this immunoregulatory mechanism, in addition to its hormonal function, it also contributes, at least in part, to improving bone mineral density [84,85].

Moreover, vitamin D’s immunoregulatory role extends beyond the mere downregulation of pro-inflammatory cytokines via specific functional suppression of Th1 and Th17 cells. It also includes the upregulation of osteoprotective cytokines through the induction of Tregs and Th2 cells. Although the specific roles remain controversial and dependent on individual inflammatory contexts and hormonal influences, vitamin D’s osteoprotective effects are further enhanced by the emerging functional synergy with the alarmin IL-33. This synergy promotes the Th2 response and inhibits the Th1 response in bone, alongside the induction of Tregs [87].

#### 3.2.2. Role of Gut Microbiota

In addition to vitamin D deficiency, a crucial etiopathogenetic contribution to OP is also offered by specific changes affecting beta diversity, taxonomy, and functional composition of the GM. In a large population-based study conducted by Wen Ling et al., a positive correlation was specifically identified between OP and the relative abundance of *Actinobacillus*, *Blautia*, *Oscillospira*, *Bacteroides*, and *Phascolarctobacterium*. Conversely, an inverse correlation with the disease was observed for *Veillonellaceae*, *Collinsella*, and *Ruminococcaceae* [91]. Similarly to what was discussed for vitamin D, GM is essentially involved in a metabolic and immunoregulatory role at the bone level. From a metabolic standpoint, the GM also ensures optimal intestinal absorption of nutrients, essential for maintaining bone health, through its metabolic products derived from the fermentation of dietary fibers, such as SCFAs [77]. Moreover, microbiota and vitamin D mutually influence each other metabolically. On one hand, intestinal dysbiosis alters calcium and vitamin D reabsorption at the intestinal level, while on the other hand, vitamin D deficiency itself induces intestinal dysbiosis through the decrease in the ratio of *Firmicutes* to *Deferribacteres* in the gut and consequent intestinal inflammation [8].

Additionally, in the physiopathology of postmenopausal OP, the bidirectional influence between GM and estrogens is also noteworthy. Specifically, on one hand, estrogen deficiency induces high levels of lipopolysaccharides, which are associated with gut dysbiosis, thus increasing the luminal *Firmicutes/Bacteroidetes* ratio. This dysbiosis appears to play a key etiopathogenic role in triggering bone inflammatory pathways, as evidenced by the improvement in bone parameters observed in ovariectomized rats following GM depletion induced by antibiotic therapy. On the other hand, reduced GM diversity is thought to be responsible for the finding of lower serum estrogen levels. This hormonal deficit is caused by impaired microbial secretion of β-glucuronidase, a key enzyme for the conversion of estrogens into their active form [77,92]. Regarding GM’s immunoregulatory role, it is known that specific dysregulations of GM influence bone immune homeostasis and thus bone remodeling, potentially triggering both gut-mediated bone inflammation through the involvement of pro-inflammatory cytokines and a deficit in osteoprotective immunity. In both cases, this results in an immune imbalance towards osteolytic, thus pro-resorptive, activity. A clear example in OP is the depletion of *Clostridium* strains, leading to suppression of Tregs in the bone. Similarly, SCFAs themselves exert an immunoregulatory action in a bone-protective sense, both by inhibiting osteoclast differentiation and by promoting Tregs differentiation, as seen with butyrate acting synergistically with parathyroid hormone (PTH). Based on such inter-kingdom communications in OP, which are further influenced by different dietary patterns, the novel concept of “Osteo-Microbiology” has emerged [8,77,93,94].

A recent Mendelian randomization analysis has confirmed a substantial etiopathogenetic link between GM and OP, identifying the causal relationship between specific GM taxa (211 in total) and OP. Specifically, an abundance of the *Pasteurellaceae* family has been associated with an increased risk of OP, whereas a reduced osteoporotic risk has been associated with the presence of the *Oxalobacteraceae* family. Mechanistically, the increased risk of OP appears to be linked to the ability of *Pasteurellaceae* to both promote bone resorption by stimulating osteoclastic activity and, through the release of specific microbial metabolites, induce apoptosis in bone cells and interfere with normal cellular signaling pathways. On the other hand, in a still entirely speculative manner, *Oxalobacteraceae* may regulate bone health through dietary patterns that remain poorly defined [95]. Similarly, another Mendelian randomization study has identified the *Burkholderiales* order as a specific microbial group correlating with an increase in osteoclasts and a reduced risk of postmenopausal OP [96].

Over the last few years, considerable interest has been growing to evaluate the protective role of the *Lactobacillus* genus in OP [97]. Specifically, it is emerging that *Lactobacillus* species, or their metabolites and/or structural components, act as potent controllers of bone cellular immunity, thereby playing a regulatory role in bone turnover and promoting bone health. Indeed, SCFAs (butyrate and propionate) derived from *Lactobacillus* spp. induce the proliferation of mature Tregs, resulting in increased osteoblast differentiation and reduced osteoclastogenesis. Moreover, Tregs activation is crucial for PTH-stimulated calciotropic bone formation [98].

A recent study in ovariectomized rats highlighted the role of *Lactobacillus rhamnosus GG* in significantly improving the Th17/Treg balance in both bone and intestine, thus attributing a protective role to this probiotic in estrogen deficiency-induced OP [99]. Interestingly, in glucocorticoid-induced OP (the most frequently encountered secondary form of OP), the osteoprotective role of *Lactobacillus plantarum* has recently emerged due to its effective promotion of intestinal microbial diversity in favor of beneficial bacteria, along with its ability to significantly increase serum levels of pyrazine and gamma-glutamylcysteine, which are involved in inhibiting osteoclastogenesis and promoting osteoblastogenesis [100]. Similar osteoprotective effects have also been observed in an in vitro study on serum valeric acid, a microbial metabolite causally downregulated by *Bacteroides vulgatus* in OP [101].

#### 3.2.3. Role of MiRNAs

Epigenetics, based on circular RNA (circRNA)–miRNA–mRNA networks and lncRNA–miRNA networks, plays a key role in the pathogenesis of OP. CircRNAs, as endogenous noncoding RNAs (ncRNAs), represent the “primum movens” in the sophisticated modulation of gene expression, subsequently involving miRNAs in bone regulation. In the clinical setting, understanding these mechanisms and the molecular processes involved is paving the way for new diagnostic, prognostic, therapeutic, and preventive frontiers [102,103,104,105,106,107,108,109,110,111,112].

An exemplary case is the potential use of highly expressed bone-metabolism-related serum miRNAs such as miR-144-5p, miR-506-3p, miR-8068, miR-6851-3p, miR-148a, and miR-122-5p as valid predictive biomarkers for diagnosing postmenopausal OP. Moreover, these could also be employed independently of the findings from traditional radiological and laboratory tests [113,114].

MiRNAs play a crucial role in bone metabolism, being involved in regulating the cellular processes of osteoblasts, osteoclasts, and osteocytes, in addition to functioning as mediators between these cells. In OP, the pathophysiological interplay between dysregulated miRNA expression, OS, and inflammation is crucial in promoting osteoclastic differentiation at the expense of osteoblastic differentiation. Given these premises, miRNAs can promote or suppress the inflammatory response and/or ROS production. Conversely, ROS and pro-inflammatory cytokines can up- or down-regulate miRNA expression in a perpetual vicious cycle [115].

An example of pathogenesis is the activation of NF-kB by TNF-α, which promotes the expression of miR-705, which, in turn, downregulates forkhead box protein O1 (FOXO1), a key protein in bone defense mechanisms against oxidative damage. Similarly, TNF-α, by upregulating miR-182, which in turn inhibits FOXO3, promotes osteoclastogenesis. On the other hand, osteoclastogenesis in postmenopausal OP is also promoted by miR-128 through the downregulation of Sirtuin1, an NF-kB inhibitor. Likewise, miRNA-141 downregulates TGF-β, resulting in a pro-inflammatory effect. Moreover, the overexpression of miR-320a in OP is correlated with altered osteoblastic differentiation and simultaneous increased ROS production [115,116,117].

Moving away from the aforementioned pathophysiological interplay, miR-15b is also involved in the worsening of OP through its direct inhibitory action on osteoblastic differentiation [118].

On the protective side, a recent in vitro and in vivo study on models of OP related to estrogen deficiency or aging identified miR-1224-5p as a key regulator of osteogenesis, both through the downregulation of RANKL-induced osteoclastic differentiation and through the simultaneous promotion of osteoblastic differentiation via the Ras-associated protein-1 (Rap1)-signaling pathway [117].

An even greater number of miRNAs recently revisited or investigated are emerging as key regulators of osteoclastic and osteoblastic differentiation processes, as well as regulators of collagen synthesis. Specifically, essential regulators of bone remodeling include miR-100-5p, miR-128, miR-185, miR-185-5p, miR-124, miR-451a, miR-133a, miR-33-5p, miR-25-3p, miR-21, miR-1-3p, miR-125a-5p, miR-146a, miR-195, miR-103a, miR-139-5p, miR-137, miR-194, miR-182, miR-183, miR-155, miR-29b, miR-99b-5p, miR-542-3p, miR-30a-3p, miR-451a, miR-1-3p, miR-210, miR-21-5p, miR-31, miR-221-5p, miR-25-3p, miR-125b, miR-486-5p, miR-582-3p, miR-320e, miR-494-3p, miR-1270, miR-23b-3p/miR-885, miR-140-3p, miR-885, and miR-494-3p [102,119,120,121,122,123].

Interestingly, a recent research focus has emerged regarding the regulatory role of miRNAs in the formation of H-type vessels, unique bone microvessels that play a crucial role in osteogenesis–angiogenesis coupling. A decrease in H-type endothelial cells has been found in experimental models of various forms of OP, including postmenopausal and aging-related OP, suggesting a key involvement of these microvessels in OP pathogenesis. Osteogenesis–angiogenesis coupling is regulated by cellular signaling pathways such as hypoxia-inducible factor-1α (HIF-1α)/vascular endothelial growth factor (VEGF), Notch, and Wnt/β-catenin signaling pathways. Some miRNAs (e.g., miRNA-7b, miRNA-49~195, miRNA-136-3p, miRNA-143, miRNA-149-5p, miRNA-188-3p, miRNA-214-3p, miRNA-375) have been found to intervene in these signaling processes, promoting or inhibiting H-type vessel formation [124].

Lastly, but no less important in the pathogenesis of OP, is the influence of GM on epigenetics. This epigenetic regulation, which occurs along the microbiota–bone axis, is based on the mechanistic premise that various non-coding RNAs can be systemically transported via extracellular vesicles to various distant cells and tissues, including the skeleton [2]. Bacterial extracellular vesicles (BEVs) are structurally characterized by a phospholipid bilayer and can be released by various bacteria, serving as a primary transport system for diverse molecules, including nucleic acids. Moreover, the advantages offered by their nanostructure, excellent biocompatibility, low toxicity, and ease of large-scale production make them promising platforms for engineering therapies targeting OP [125].

**Figure 2:**
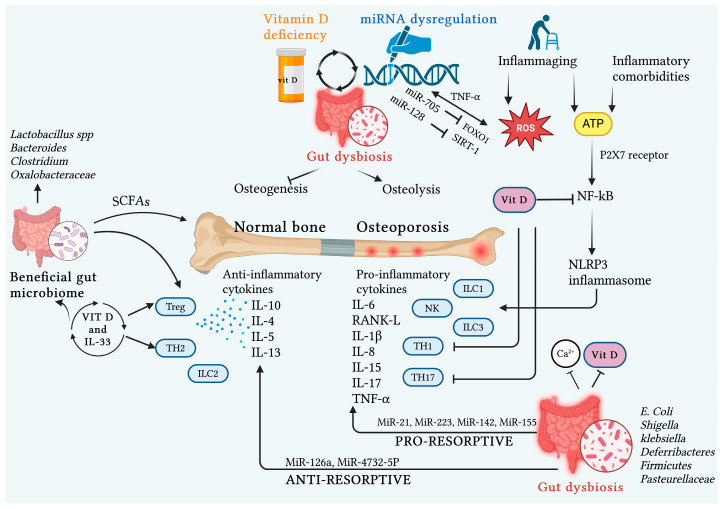
On the right, depiction of the etiopathogenic influence on osteoporosis driven by the interplay among vitamin D deficiency, gut dysbiosis, and miRNA dysregulation. Specifically, epigenetic dysregulation, together with inflammaging and other systemic chronic inflammation substrates, promotes oxidative stress and activates NF-kB as a pathway involved in cell activation and subsequent pro-inflammatory cytokine release. Concurrently, gut dysbiosis contributes to bone inflammation by both impairing vitamin D absorption and releasing pro-resorptive miRNAs. On the opposite side of the image, the immunoregulatory synergy established between beneficial gut microbiota derived-SCFAs and the optimal vitamin D/IL-33 crosstalk promotes Tregs, Th-2, and ILC2 cells, which are key players in supporting bone health through the release of anti-inflammatory cytokines. Abbreviations: innate lymphoid cells (ILCs), short chain fatty acids (SCFAs), regulatory T cells (Tregs), T helper cells (TH), natural killer cells (NK), interleukin (IL), nuclear factor kappa-light-chain-enhancer of activated B cells (NF-kB), reactive oxygen species (ROS), adenosine triphosphate (ATP), NLR family pyrin domain containing 3 (NLRP3), tumor necrosis factor (TNF). Created with BioRender.com.

Specifically, regarding miRNAs, in a context of OP-related gut dysbiosis, various microbial species exert their epigenetic regulatory role in an osteoprotective or osteoresorptive direction. In this regard, *Firmicutes* and *Klebsiella pneumoniae* are capable of promoting the expression of miRNAs primarily involved in OP genesis as pro-inflammatory and bone-resorptive agents, such as miR-21 (*Firmicutes*) and miR-142 and miR-223 (*Klebsiella*). Similarly, *Lactobacillus acidophilus* and *Bifidobacterium bifidum* upregulate miR-155, which has a pro-inflammatory role via increased TNF-α, RANK, IL-1beta, macrophage colony-stimulating factor (M-CSF) and B-cell lymphoma (Bcl)-2, thereby promoting bone resorption and inhibiting osteogenic differentiation. Conversely, *E. coli* and *Shigella* are among the microbial species challenging OP due to their ability to increase the expression of osteoprotective miRNAs, promoting osteogenesis, such as miR-146a and 4732-5p, respectively [8] (Figure 2) (Table 1).

### 3.3. Skin–Bone Axis: The Pathophysiological Fil Rouge Between Skin Disorders and Osteoporosis

#### 3.3.1. From Skin to Bone

The strong clinical link between osteoporosis (OP) and chronic or autoimmune inflammatory skin diseases arises from two main factors. First, both conditions share a common pathophysiological basis in chronic inflammation, characterized by distinct cytokine patterns that promote osteoclastogenesis. Second, systemic treatments commonly used for skin disorders, such as glucocorticoids and immunosuppressants, significantly increase the risk of developing OP [2].

This link has been highlighted by Mizutani et al., who, in a murine model of persistent dermatitis, identified a key pathogenic role of skin-derived pro-inflammatory cytokines (such as TNF-α and IL-6) in accelerating the onset of secondary OP [126].

This is also confirmed by recent literature reviews suggesting an increased risk of osteopenia and OP in patients with chronic and extensive psoriasis, whether or not associated with psoriatic arthritis. This is based on the assumption that pro-inflammatory Th1 cytokines (primarily TNF-α and IL-6) involved in the formation of psoriatic plaques are also stimulators of osteoclastogenesis in the bone. Specifically, TNF-α promotes bone resorption either directly or by stimulating the expression of RANKL and macrophage colony-stimulating factor (M-CSF) in osteoblasts, stromal cells, and osteocytes. Synergistically with RANKL, TNF-α thus supports osteoclastic differentiation via the NF-κB pathway. Similarly, IL-6, as a pleiotropic pro-inflammatory cytokine, acts at the bone level by promoting osteoclastogenesis, inhibiting osteoblastic differentiation, and facilitating the secretion of pro-osteoclastic mediators such as RANKL, IL-1, PTH-related protein, and prostaglandin E2 (PGE2). In this context, the role of oncostatin M (OSM), a cytokine belonging to the IL-6 family and upregulated in chronic inflammatory skin diseases, remains controversial, as it has both anabolic and catabolic effects on bone. In fact, preliminary evidence supports, on one hand, the anabolic effect of OSM on bone by directly promoting osteoblastic differentiation. On the other hand, the multifunctional nature of this cytokine appears to be explained by its promotion of bone loss through increased RANKL expression. Moreover, IL-17 also plays a key pathogenic role in bone loss induced by psoriatic inflammation.

Similarly, drugs used in psoriasis act as both a blessing and a curse in the development of OP. Immunosuppressants like methotrexate and cyclosporine A negatively affect bone metabolism by inhibiting osteoblast differentiation. Specifically, the deleterious effects of methotrexate on bone have not yet been extensively studied, with only limited in vitro evidence suggesting its role in attenuating osteoblast precursor differentiation and, consequently, bone formation. In contrast, more robust evidence supports the causal role of cyclosporine A in inducing severe and rapid trabecular bone loss by inhibiting osteoblastic differentiation through the calcineurin–nuclear factor of activated T cells (NFAT) signaling pathway. For these reasons, especially when combined with systemic corticosteroids, which impair collagen synthesis, these drugs contribute to bone loss and promote osteoporosis. On the other hand, therapeutic approaches such as phototherapy exert anti-osteoporotic effects by increasing vitamin D levels. Promising in this regard are also IL-17 neutralizing antibodies [127,128].

Furthermore, the crosstalk between IL-33 and vitamin D plays a key role in the pathogenesis of OP associated with psoriasis. Mechanistically, evidence suggests that vitamin D and IL-33 act synergistically in certain biological processes, while in other cases, they functionally influence each other. This close functional relationship is mediated through the sharing of specific signaling pathways and plays a crucial role in maintaining bone and skin homeostasis. IL-33 recognizes ST2 as its primary receptor, which exists in two distinct forms: a transmembrane receptor acting as a cellular receptor, and a soluble form (sST2) functioning as a decoy receptor, thus inhibiting IL-33 activity. Given the presumed protective role of IL-33 at the skeletal level, physiologically, this crosstalk would be involved in promoting anti-osteoclastogenic cytokines such as IL-4 and IL-10 through the stimulation of both Tregs and Th2 cells. However, in a context of comorbidity between psoriasis and OP, and shared pathophysiological mechanisms such as vitamin D deficiency and inflammatory pathways (particularly RANKL-mediated), the IL-33/ST2 axis exhibits a paradoxical effect, shifting from an osteoprotective to a proinflammatory and osteoclastogenic role, thereby driving the development of OP. This detrimental process involves several mechanisms, including PTH hyperproduction, impaired Treg function, decreased sST2 expression, and increased Th1 and Th17 activity, thus leading to the production of proinflammatory osteoclastogenic cytokines [87].

In addition to psoriasis, the clinical association with OP is also documented for other major autoimmune and chronic inflammatory skin diseases, such as chronic spontaneous urticaria (CSU), AD, vitiligo, immunobullous diseases, and seborrheic dermatitis. Regarding CSU, the pathophysiological substrate appears to be related to the ability of MC degranulation products to increase bone resorption at the expense of bone formation. Mechanistically, MC mediators such as IL-6, C-reactive protein, D-dimer, fibrin, and degranulation products activate the RANKL/RANK/osteoprotegerin (OPG) pathway, thereby promoting the differentiation of macrophages into pre-fusion osteoclasts, multinucleated osteoclasts, and ultimately activated osteoclasts. On the other hand, the association between AD and OP is not due to a common inflammatory substrate but is mainly justified by the osteoresorptive effects of immunosuppressants such as cyclosporine, as well as by the severe malnutrition resulting from avoidance diets undertaken by AD patients for suspected food allergies. Regarding cyclosporine, preliminary evidence from murine models suggests its role in increasing bone turnover. In this context, it exerts an osteolytic effect by reducing bone volume, decreasing the number and thickness of trabeculae, and further increasing trabecular separation. The clinical association between vitiligo and OP is similarly attributed to the shared influence of pro-inflammatory cytokines, such as IL-17, IL-6, and IL-2, which affect bone remodeling [2].

Similarly, and independently of long-term systemic corticosteroid use, the association between pemphigus (and generally autoimmune blistering skin diseases) and OP is based on the shared chronic pro-inflammatory substrate of cytokines such as IL-17, TNF, IL-1, and IL-6, which, together with auto-reactive B and T cells, drive osteoclast-mediated bone resorption. Additionally, other non-steroidal causes of bone mineral density loss in these patients include reduced physical activity and malnutrition, leading to poor production of insulin-like growth factor-1, crucial for stimulating osteoblastic activity. Interestingly, vitamin D deficiency appears to be a characteristic pathophysiological trait in patients with pemphigus, with vitamin D levels inversely correlated with disease activity. This leaves open the hypothesis, deserving of further investigation, of bone loss not as a separate pathophysiological event but as another manifestation of vitamin D deficiency related to pemphigus [129].

Remaining in the realm of chronic skin fragility disorders and their clinical correlation with OP, a significant link has also been recognized between postmenopausal bone mineral density alteration and skin aging-related changes, such as reduced dermal thickness, decreased skin elasticity, and reduced skin collagen production. In this sense, the alteration of a skin parameter could potentially predict the alteration of a bone parameter [130].

A recent cross-sectional study found a high prevalence of dermatoporosis in patients with OP [131]. Both diseases in their primary forms are clinical expressions of the aging process at the skin and bone levels, respectively, and share the pathophysiological substrate of vitamin D deficiency. Indeed, adequate levels of vitamin D act as an essential immunoregulator of the inflammaging process in both tissues, as well as promoting collagen synthesis and maintaining optimal structural integrity of bone and skin [132].

Even more interestingly, age-related bone loss and thus the onset of senile OP would be directly influenced by skin aging through the reduced secretion of cystatin-A. At the cutaneous level, this keratinocyte-derived hormone, expressed in the epidermal stratum corneum, prevents epidermal barrier dysfunction by exerting an inhibitory effect on exogenous proteases. A loss of its function in the skin has been associated with the onset of various dermatoses, such as AD, acral peeling skin syndrome, and autosomal recessive exfoliative ichthyosis. At the bone level, in non-aged skin, by binding receptor for activated C-kinase 1 in osteoblastic and osteoclastic progenitors, cystatin-A physiologically promotes osteoblastic differentiation while blocking osteoclastic differentiation. This crucial pathophysiological mechanism is indirectly confirmed by the topical application of calcipotriol, which, by stimulating the epidermal production of cystatin-A in aged skin, attenuates OP [133].

A recent population-based retrospective cohort study highlighted for the first time a clinical association between seborrheic dermatitis and OP, particularly in female and young age groups with comorbidities such as hyperthyroidism, hyperlipidemia, and epilepsy. This association is based on the shared pro-inflammatory substrate of the conditions, primarily involving osteoclastogenic cytokines such as RANKL, TNF-α, and IL-6 [134].

#### 3.3.2. From Bone to Skin: An Emerging Dichotomy

**Figure 3:**
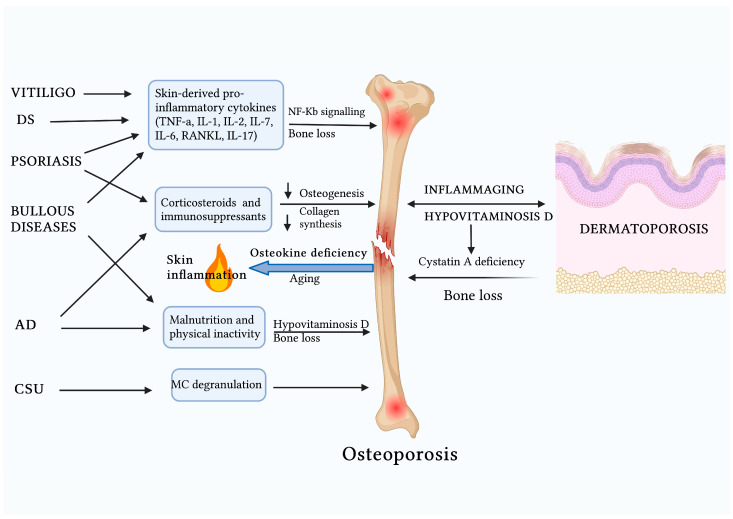
Representation of the bidirectional pathophysiological interactions between chronic autoimmune/inflammatory skin diseases and osteoporosis within the skin–bone axis. From skin to bone, bone loss is driven by systemic effects of skin-derived pro-inflammatory cytokines, which in turn promote NF-kB-related pro-inflammatory signaling. Additionally, bone loss in certain skin disorders is exacerbated by factors such as corticosteroid and immunosuppressant use, malnutrition, and physical inactivity, leading to reduced osteogenesis, collagen synthesis, and vitamin D deficiency. Furthermore, in the context of skin and bone fragility, clinically expressed in osteoporosis and dermatoporosis—both associated with inflammaging and vitamin D deficiency—the resulting cutaneous cystatin A deficiency impacts bone, causing further loss. From bone to skin, aging-related osteokine deficiency is a primary pathophysiological event that would trigger skin inflammation. Abbreviations: seborrheic dermatitis (DS), atopic dermatitis (AD), chronic spontaneous urticaria (CSU). Created with BioRender.com.

From what has been discussed so far, the notion of skin as a “mirror” of internal organ health, including that of the skeleton, clearly emerges. However, even more interesting in the context of the skin–bone axis is the bidirectional pathogenetic crosstalk that is established. This reciprocal exchange of endocrine and metabolic signals is the balance point between the health and disease of the two organs. Moreover, while the pathophysiological influence of the skin on the bone via systemic inflammation, drugs, and vitamin D deficiency is more obvious and extensively investigated, the opposite influence has been less discussed in the literature. In this context, a primary pathophysiological role is assigned to osteokines of osteoblastic origin such as osteocalcin (OCN), fibroblast growth factor-23 (FGF23), and lipocalin-2 (LCN-2). These, acting as true hormones, physiologically ensure homeostatic balance between various organs. For example, FGF23 can regulate systemic phosphate and vitamin D levels. Specifically, these osteokines influence skin health through anti-inflammatory and anti-apoptotic effects, as well as by regulating skin barrier function. Serum levels of these osteokines have been investigated and correlated with psoriasis. Furthermore, their serum levels (particularly of OCN) decrease with aging. Based on these premises, a presumed anti-inflammatory role of these osteokines at the skin level would justify the pathophysiological influence of aged bone on the onset of chronic inflammatory skin disorders [1] (Figure 3).

## 4. Conclusions and Perspectives

From the discussion so far, it is clear that skin and bone should be considered distinct organs only from an anatomical perspective. Functionally, however, they share immunoendocrine mechanisms that act as a balancing point between health and disease in both organs. Clinically, this bidirectional immunoendocrine communication is most evident in the well-documented association between OP (which can be regarded as an inflammatory condition) and major chronic autoimmune/inflammatory diseases or skin fragility disorders. Therefore, within the context of the recognized skin–bone axis, the skin reflects the health status of the bone and, conversely, the bone mirrors the health status of the skin. The etiopathogenetic bidirectionality of this axis is governed by pro-inflammatory pathophysiological mechanisms involving various cytomolecular players, including skin-derived cytokines and bone-derived cytokines (osteokines). In such a bidirectional framework, aging and the associated low-grade chronic inflammation (inflammaging) are well-established influencing factors. Additionally, an emerging etiopathogenetic role can be attributed to the pathophysiological interplay involving gut dysbiosis, vitamin D deficiency, and miRNA dysregulation, which can be considered the three common denominators of both skin and bone inflammatory responses. This interplay affects two crucial physiological mechanisms: redox balance and immune homeostasis. The resulting imbalance leads to increased ROS production and organ-specific inflammation, which reciprocally influence each other. The influence of the discussed pathophysiological interplay on the dysregulation of two key biological mechanisms becomes particularly evident in vitiligo. Here, vitamin D deficiency, through the downregulation of antioxidant pathways, leads to increased ROS production. This process is further supported by a specific state of gut dysbiosis, characterized by reduced Bacteroidetes and Lachnospiraceae, and by the upregulation of specific miRNAs (miR-25, miR-9, miR-377, and miR-493-3p), which further promote ROS production in melanocytes, ultimately leading to immune dysfunction and their destruction.

Moreover, the impaired control of OS caused by vitamin D deficiency also results in poor regulation of chronic systemic inflammation, a crucial factor in the development of various OS-mediated age-related disorders, including OP. In a vicious cycle, miRNAs can either promote or suppress inflammation and ROS production. Conversely, ROS and pro-inflammatory cytokines can upregulate or downregulate specific miRNAs.

In OP, the interplay between dysregulated miRNAs, OS, and inflammation drives osteoclastic differentiation at the expense of osteoblastic one, ultimately promoting bone loss.

As part of this complex cytomolecular network, a key signaling hub is represented by the NF-κB pathway, which is upregulated by vitamin D deficiency and epigenetic dysregulation in both OP and the examined skin disorders. Indeed, vitamin D exerts a physiological inhibitory effect on this signaling pathway in both skin and bone. In the absence of this inhibitory effect due to vitamin D deficiency, NF-κB becomes upregulated and drives organ inflammation. In parallel, specific epigenetic dysregulations contribute to this upregulation. In psoriasis, miR-31 plays a key pro-inflammatory role via NF-κB.

On the flip side, miR-9, downregulated in psoriasis, has an anti-inflammatory role through the downregulation of NF-κB. Moreover, miR-146a is upregulated by NF-κB in the later stages of inflammation and acts through a negative feedback mechanism to suppress this signaling. In AD, miR-124 (downregulated) is thought to exert anti-inflammatory effects by inhibiting p65, a subunit of NF-κB. In postmenopausal OP, miR-128, through the downregulation of Sirtuin1, an inhibitor of NF-κB, promotes NF-κB signaling, which is involved in osteoclastogenesis.

The interplay is further completed by gut dysbiosis, which impacts both bone and skin through the reduced production of SCFAs and consequent immunoregulatory deficit, as well as through its epigenetic influence, which can be either pro- or anti-inflammatory. Specifically, at the cutaneous level, SCFAs exert their immunoregulatory role both by controlling Th17-mediated inflammation and by regulating the release of Th2 cytokines such as IL-4, IL-5, and IL-13. Consequently, in the context of the discussed dermatoses, a dysbiosis-mediated SCFAs deficit plays a key etiopathogenetic role in the onset of psoriasis and AD.

At the bone level, the immunoregulatory—and thus osteoprotective—role of SCFAs, such as butyrate and propionate, is specifically exerted by inhibiting osteoclastic differentiation and promoting Tregs differentiation. Thus, in the context of a dysbiosis-mediated SCFAs deficiency, impaired Treg function, together with enhanced osteoclastogenesis, promotes bone loss and consequently the onset of OP.

Our narrative approach underscores the significant clinical potential that could emerge from an increasingly detailed characterization of the intricate and reciprocal interactions among vitamin D, miRNAs, and GM in two conditions with high epidemiological and socioeconomic relevance: OP and chronic autoimmune/inflammatory skin diseases. Particularly promising is the potential of a triple therapeutic strategy, combining traditional vitamin D supplementation with pioneering GM-targeted interventions and epigenetic manipulation techniques leveraging agomir/antagomir technologies. To make such an approach clinically feasible, some current limitations must be overcome, including the following:

(1) The need for stricter regulatory oversight by drug agencies regarding the use of current pre- and probiotics, whose production is still largely prerogative of individual nutraceutical laboratories.
(2) A more rigorous characterization of the expression and functional profiles of miRNAs (miRNome analysis) in both conditions.

In this context, the identification of common patterns of epigenetic dysregulation would have significant diagnostic and therapeutic implications. On the diagnostic front, the use of shared upregulated or downregulated miRNAs as predictive biomarkers could pave the way for early diagnosis and more effective follow-up of these comorbidities. On the therapeutic side, within the framework of a redefined concept of “One size fits all,” such insights could lead to the use of a single drug (agomir or antagomir) to treat organ-specific diseases that are anatomically distant but mechanistically linked. Therefore, a close multidisciplinary collaboration involving orthopedists, physiatrists, immunorheumatologists, gastroenterologists, and dermatologists to design clinical studies elucidating the fundamental mechanisms of the skin–bone axis is highly advisable. The current clinical setting would benefit from a multidisciplinary approach primarily aimed at the early identification and risk stratification of OP in dermatological patients undergoing treatment with high-potency topical or systemic corticosteroids or immunosuppressants. Continuous monitoring of laboratory parameters (vitamin D, calcium, phosphorus, and PTH) and instrumental markers (BMD) related to bone remodeling would encourage the design of clinical trials to assess the safety and therapeutic efficacy, on both cutaneous and bone inflammation, of targeted biological therapies (e.g., anti-TNF, anti-IL-6, anti-RANKL, and anti-IL-17) in such patients.

Similarly, involving gastroenterologists in this network would pave the way for clinical trials evaluating the efficacy of probiotic or fiber-rich dietary regimens, as well as supplementation with pre- and probiotics or their metabolites, such as SCFAs, in patients with these comorbidities. A one-size-fits-all approach in combinatorial therapy holds promising prospects for the future.

## Figures and Tables

**Table 1:** Schematization of the pivotal cytomolecular events and interlinked pathophysiological mechanisms induced by hypovitaminosis D, gut dysbiosis, and epigenetic dysregulation in chronic inflammatory skin disorders and in osteoporosis. Abbreviations: T helper cells (Th), forkhead box P3 regulatory T cells (FoxP3 Tregs), SCFAs (short chain fatty acids), reactive oxygen species (ROS), oxidative stress (OS), interleukin (IL), tumor necrosis factor α (TNFα), nuclear factor kappa-light-chain-enhancer of activated B cells (NF-κB).

Skin Disorders	Hypovitaminosis D	Gut Dysbiosis	Epigenetic Dysregulation
**Vitiligo**	Decreased immune tolerance ↓ Increased release of pro-inflammatory cytokines	Decrease in *Bacteroidetes* and *Lachnospiraceae* ↓ ROS production ↓ Immune dysfunction and melanocyte destruction	- MiR-21 downregulation Tregs/Teffector → imbalance - MiR-9/25/377/493-3p upregulation →ROS production
**Psoriasis**	Increased IL-12/23, TNFα, Th-17 driven inflammation	Decrease in *Faecalibacterium*, *Akkermansia muciniphila*, *Bifidobacterium*, *Lactobacillus* vs. Increase in *E. coli* and *Ruminococcus gnavus* ↓ SCFAs downregulation ↓ Th-17 driven inflammation	- MiR-31/155/210 upregulation → FoxP3 Tregs and Th-2 suppression - MiR-9/146a downregulation →Th-17 driven inflammation
**Alopecia Areata**	Decreased immunomodulation	Increase in *Erysipelotrichaceae* and *Clostridiales* ↓ Immune activation and follicular destruction	- HOTAIR downregulation →NF-κB signalling activation ↓ IL-1b, TNFα driven inflammation ↓ Hair growth inhibition
**Atopic Dermatitis**	- Th-1/Th-2 imbalance ↓ IL-4/5/6/13—oriented inflammation - LL-37 downregulation ↓ *S. Aureus* colonization	Decrease in *Lactobacillus rhamnosus*, *Faecalibacterium*, *Bifidobacterium* ↓ SCFAs downregulation ↓ IL-4/5/6/13—oriented inflammation	- MiR-155/10a-5p/29b/939 upregulation →Th-17 differentition, epithelial barrier dysfunction, *S. Aureus* colonization - MiR-124 downregulation →NF-κB signalling activation
**Immunobullous Blistering Skin Disorders**	Decreased immunomodulation ↓ Tregs and B cell suppression, Th-17 driven inflammation	Decrease in *Lachnospiraceae*, *Veillonellaceae*, *Prevotellaceae*, *Faecalibacterium* vs. Increase in *Bacteroidaceae*, *Ruminocoaccaceae*, *Enterobacteriaceae* ↓ Immune dysfunction	- MiR-338-5p upregulation →NF-κB signalling inhibition
**Osteoporosis**	NF-κB signalling activation and OS promotion ↓ Upregulation of pro-inflammatory osteoclastogenic cytokines (via Th-1 and Th-17 activation) and downregulation of osteoprotective cytokines (via Tregs and Th-2 suppression)	Increase in *E. coli*, *Shigella*, *Klebsiella*, *Deferribacteres*, *Firmicutes*, *Pasteurellaceae* → impaired intestinal absorption of vitamin D →Pro-resorptive miRNAs upregulation ↓ Osteoclastogenic cytokines activation	- MiR-128/705 upregulation ↓ROS production and NF-κB signalling activation - Gut dysbiosis related-MiR- 21/142/155/223 upregulation →pro-inflammatory osteoclastogenic cytokines activation

## Data Availability

Not applicable.
